# Brightening Orthodontic Appliances

**Published:** 1907-12-15

**Authors:** W. J. Brady


### BRIGHTENING ORTHODONTIC APPLIANCES.

BY W. J. BRADY, D. D. S.

The oxide after soldering is often difficult of removal from appliances, especially for gold plating, where every vestige of oxide, flux and discoloration must be removed, and the metal left clean and bright. It is tedious to remove this oxide by the ordinary process of polishing, and delicate bands or other small parts are likely to be badly twisted out of shape. Where metals are prepared for plating in wholesale quantities, as in jewelry factories, the oxide is removed and the metal made bright entirely through the use of "dips" or "pickles."

Different metals require different treatment. What will give a clean, bright surface on one will sometimes have little effect on another, or it may act so energetically as to dissolve it altogether. It would be a long story to give the best treatment for all the different metals, and it requires much experience to handle successfully an assortment of metals, including copper, brass, oreide, "red metal," silver, aluminum, bronze, gold, nickel silver and platinum, and have the same come from the various baths clean and bright.

German silver, or nickel silver, as it is often called, may be cleaned by boiling for a few moments in a pickle composed of:

| | |
|---|---|
| Sulphuric acid | 3 parts. |
| Nitric acid | 1 part. |
| Water | 10 parts. |

This pickle should be kept in a glass-stoppered bottle, properly labeled. The most convenient method of using is to pour enough pickle in a large test tube to cover the work and hold the same in a Bunsen or other flame. When hot, the work (previously strung on copper or brass wire) is dipped in, boiled just long enough to brighten the metal, and then removed at once and dipped into clean water, after which it is plunged quickly into a mild solution of common cooking soda.

The action of the hot pickle is quite energetic, and several minutes boiling would dissolve thin bands, the nitric acid acting on the zinc of nickel silver very quickly. It is very important not to overdo the boiling. Usually the oxide is thoroughly removed in ten to twenty seconds.

The fumes from this pickle are irritating, and should be kept from spreading over the office as much as possible. It is well to do the boiling near an open window, where convenient. The bottle must be kept tightly stoppered, and the pickle not kept in an open vessel like the commonly used sulphuric acid pickle for gold, or the fumes constantly arising will rust all the steel instruments within reach, and certainly would do no particular good to anything else about the place.

Appliances containing considerable copper, as bronze, oreide, "goldine," etc., can be brightened by use of a cyanide dip. This is composed of:

| | |
|---|---|
| Potassium cyanide | 1 part. |
| Water | 10 parts. |

The work is strung on a wire and allowed to remain in this solution several minutes, when it becomes bright. If much discolored by soldering, it is better to boil a few seconds in the acid pickle first given, then after a thorough rinsing and dip in the soda solution and second rinsing, it may be transferred to the cyanide dip.

Potassium cyanide is a deadly poison, and must be used with great care. The solution should be kept in a wide-mouthed glass-stoppered bottle, that the work may be dipped into the original receptacle, and the dip need not be poure out into glasses, dishes, etc., that might accidentally be used for drinking purposes. The bottle should be plainly labeled, and have a glaring skull and crossbones with "POISON!" plainly marked. This solution may be absorbed into cuts, scratches, or any skin abrasions, and produce serious trouble. Unless it is handled with all due precaution, it had better not be used at all.

Before attempting to brighten up any metal by pickling, it must be free from all oil or greasy dirt. Even handling with the fingers leaves sufficient oily material to interfere with perfect brightening. Work should therefore be dipped in a strong alkali before placing in the pickle or brightening dip. A strong caustic potash solution is best for this, and this solution itself will help brighten the work. The work must be well rinsed after removing from the potash.

If appliances thus brightened are to be gold plated, they should be placed in the plating bath as quickly as possible after brightening, or a film of oxide will quickly gather on the surface from contact with the atmosphere. It takes but five or six minutes for German silver to oxidize over slightly, thus giving a dull luster to the metal instead of a bright one. The subsequent gold plating will be dull and lusterless in proportion. In plating, it is important to have everything ready at the start, and to transfer the work from one bath to another with no delay, beginning with the potash solution and ending with the gold bath.

Appliances often become discolored in the mouth from accumulations that either are not or cannot be removed by the tooth brush. Such appliances should be removed every few weeks and given a thorough cleansing. This can be easily accomplished by immersing them for a few minutes in a strong solution of caustic potash or caustic soda. The appliance should be gently stirred around in the solution with a glass rod or stick—metal should not be used.

A stock of this solution (say a pint) should be made up and kept in a glass-stoppered bottle. See that it is properly labeled, with a "POISON" label in addition. A few spoonfuls may be poured out into a shallow dish, as a sauce dish, and the appliance entirely covered. It will quickly remove all black deposits, leaving the appliance clean and polished like new. Should any change or additions be desired requiring soldering, there will be a welcome absence of that penetrating odor that follows the heating of an unclean appliance.

This solution can be used to good advantage in cleaning crowns or bridges previous to repair. It is customary to boil such pieces in acid, but the acid will not remove accumulations like a strong alkali, neither will it remove the discoloration of the metal itself, which becomes fixed and very hard to remove if heated. The caustic alkaline bath avoids all this trouble.

This word of caution must be issued: The appliance must be thoroughly washed before returning to the mouth, preferably by scrubbing with a brush under running water. All tubes or other enclosed parts should be well wiped out by thrusting cotton through the same, as a single drop of the caustic will make a deep and painful burn upon the delicate tissues of the mouth. Similarly the eyes should be guarded against an accidental splash when the work is dropped in the dish. The solution may be used repeatedly.—*Western Journal.*

